# Development and Characterization of Biocomposite Films Based on Polysaccharides Derived from Okra Plant Waste for Food Packaging Application

**DOI:** 10.3390/polym14224884

**Published:** 2022-11-12

**Authors:** Ibukunoluwa Fola Olawuyi, Won Young Lee

**Affiliations:** 1 School of Food Science and Biotechnology, Kyungpook National University, Daegu 41566, Korea; 2 Research Institute of Tailored Food Technology, Kyungpook National University, Daegu 41566, Korea

**Keywords:** biocomposite films, okra waste, biopolymer, packaging, tomatoes, preservation

## Abstract

Polysaccharide-based composite films were developed using mucilage polysaccharides (OLP) and carboxymethyl cellulose (CMC) extracted from okra leafstalk wastes. The rheological properties of biocomposite OLP/CMC film-forming solutions were characterized using the Power-law model, and fabricated films were characterized for their potential food packaging applications. OLP/CMC solutions exhibited pseudo-plastic fluid characteristics and differences in rheological behavior (*n*, 0.478–0.743), and flow consistency (*K*, 1.731–9.154) with increasing content of OLP (5 to 30 % *w*/*w* of CMC) were associated with variations in the physical, mechanical, and barrier properties of films. Surface hydrophobicity (24%) increased and oxygen (39%) and water vapor (32%) permeability reduced in OLP/CMC films containing up to 10% OLP. Moreover, a higher content of OLP enhanced the antioxidant activity and thermal stability of OLP/CMC films. Subsequently, OLP/CMC was applied as a coating to preserve cherry tomatoes for 14 days at 30 °C. Quality deterioration characterized by high weight loss (22%), firmness loss (74.62%), and discoloration (∆E, 21.26) occurred in uncoated tomatoes and were within unusable/unmarketable limits based on their visual quality score. In contrast, OLP/CMC effectively minimized quality losses, and coated tomatoes exceeded the limit of marketability after 14 days of storage. This study successfully applied value-added polysaccharides derived from okra plant residues for edible food packaging.

## 1. Introduction

Recently, biodegradable film packages produced from naturally derived polymers such as polysaccharides, proteins, lipids, or their mixtures as composites have been extensively reported as suitable alternatives for plastic/synthetic films [1,2,3]. These biopolymer films have gained much popularity and acceptance due to environmental friendliness, biodegradability, non-toxicity, and cost-effectiveness. Among the categories of biopolymers, polysaccharides such as carboxymethyl cellulose (CMC), pectin, gums, and mucilages have been used as edible coating or films to preserve fresh produce [4,5,6]. More interestingly, these polysaccharides could be obtained from alternative sources such as plant wastes [7]. When applied as a coating, polysaccharide solutions form a thin-film layer on the fruit surface, offer barrier properties such as gaseous and water permeability via the fruit surface, and create modified internal atmospheric conditions within the fruit, resulting in significant preservative effects [5]. On the other hand, they can also be used to fabricate flexible and ductile films such as food wraps [8]. To improve the preservative effects, barrier, and mechanical properties of polysaccharide-based films, various additives, including plasticizers (enhance mechanical properties) and essential oil (antimicrobial agent), are incorporated into their matrixes [5]. In addition, combining two or more polysaccharides as composites has been recommended to improve the important techno-functional properties of films/coatings [9,10,11].

Okra (*Abelmoschus esculentus*), a subtropical plant mainly cultivated in temperate regions, has recently been increasingly grown in South Korea [12]. The okra pods are the main edible part, and other plant parts (leaves, stalk, and stems) accumulate as residues, which constitutes environmental pollution. To valorize its wastes, polysaccharides with film-forming abilities have been obtained from okra leaves [13]. Moreover, the leafstalk, which contains a large amount of cellulose [14], could be synthesized into CMC, which is applicable for food packaging purposes. Polysaccharides derived from various plant wastes have been used to prepare edible films or coatings [15,16,17]. However, there is no study yet on the packaging application of polysaccharides derived from okra plant wastes.

Cherry tomatoes (*Solanum capsicastrum*), eaten fresh or in minimally processed form, are one of the most marketed and consumed fresh produce [18]. They have a unique color and intense flavor and are consumed for their high nutritional benefits, including antioxidant-rich carotenoids, phenolic acids and flavonoids, and vitamin E [19]. However, like other climacteric crops, they deteriorate rapidly after harvesting under inappropriate storage conditions [20]. Major environmental factors influencing quality loss include respiration, relative humidity, temperature, and microbial spoilage, which affect the quality parameters of appearance, color, texture, and taste, responsible for its economic valuation [21]. Various preservation techniques have been used to improve the storage quality of fresh whole cherry tomatoes [22]. A polysaccharide-based composite film consisting of CMC and gelatin (75:25 *v*/*v*) preserved the quality of cherry tomatoes by substantially reducing weight loss and browning index during 14 days [23]. In addition, cherry tomatoes coated with a composite film-forming solution containing chitosan and casein, functionalized with oregano essential oil, had lower weight loss and shrinkage than uncoated tomatoes, and fungal growth was inhibited during 28 days of storage at 4 °C [19]. Similar reports on the effectiveness of polysaccharide-based composite packaging, with or without incorporating antimicrobial essential oils and other active ingredients for preserving cherry tomatoes, have been documented [20,21,22,24]. In these studies, the composite polymer matrix showed improved barrier properties and better preservative effects compared to their single film-forming solutions, owing to the synergistic effect of the individual polymers in the composite matrix, which makes up for the shortcomings of the single-polymer and results in a superior performance [5,25].

Considering the abovementioned factors, this present study evaluated the packaging potentials of novel polysaccharide-based composite films derived from okra plant waste. In detail, cinnamon essential oil (CEO) was added to functionalize composite films consisting of okra leaf mucilage polysaccharide (OLP) and CMC derived from the leafstalk waste of okra plant. The important packaging parameters of films intended for preservative functions, such as opacity, wetting properties, water vapor barrier (WVP), oxygen permeability, and mechanical and antibacterial properties, were analyzed. Subsequently, composite film solutions were applied as edible coatings to preserve the quality of cherry tomatoes during storage at 30 °C for 14 days.

## 2. Materials and Methods

### 2.1. Materials

Okra leaves with stalks were collected from a local okra farm field in Danggin city, Chungcheongnam-do Province, South Korea. The leaves were separated from the stalks manually and dried in an oven until constant weight. Dried leaves and stalks were pulverized into powder (RT-04, Mill Powder Tech., Tainan, Taiwan) and stored at −22 °C in sealed polyethylene bags until use. All chemical reagents used were purchased from Duksan chemicals (Ansan, Korea). Pure organic cinnamon cassia essential oil (EO) was purchased from Cliganic Organics (CL-BE-031-1, Walnut, CA, USA).

### 2.2. Extraction of Biopolymers from Okra Plant Wastes

#### 2.2.1. Okra Leaf Polysaccharides (OLP)

OLP was extracted and characterized as described in our previous study [13]. Briefly, okra leaf powder was mixed with distilled water and extracted using an ultrasonic device (KHC-1SUMP, Kyung il Ultrasonic, Ansan, Korea). Mucilage extract was obtained by centrifugation and precipitated using three volumes of ethanol overnight at 4 °C. Polysaccharide precipitate was dialyzed (3.5 kDa) against water, lyophilized, and pulverized into powder (OLP). OLP is a pectic polysaccharide with an average molecular weight of 26.9 kDa and consists mainly of Gal, GalA, Rha, and Ara sugars in percentage molar ratios of 54%, 29%, 9%, and 5%, respectively [13].

#### 2.2.2. Synthesis of CMC from Okra Stalk

CMC was synthesized from okra stalk cellulose according to the method described by Rachtanapun et al. [26]. Cellulose was isolated from OKS by alkaline deep eutectic solvent (ADES, choline chloride, and monoethanolamine, 1:6) as described by Yang et al. [27], with modifications. OKS powder was mixed with ADES (1:10 *w*/*v*) in a capped flask and heated in an autoclave at 121 °C for 30 min. After cooling, the supernatant was separated, and the residue was washed exhaustively with distilled water. ADES was applied to completely remove lignin, hemicellulose, and extractives from OKS, leaving cellulose as the residue. The cellulose residue was bleached in 0.7% sodium chlorite solution (pH 4) and dried to obtain pure cellulose. Thereafter, bleached cellulose was alkalinized using a 30% sodium hydroxide solution, followed by etherification by adding sodium monochloroacetate to the mixture. The solid residue was washed exhaustively in methanol, neutralized using acetic acid, and dried to obtain CMC. More details on the synthesis and structural characteristics of OKS-derived CMC are available in the supplementary file (Figures S2 and S3).

### 2.3. Preparation of Composite OLP/CMC Films

Composite films were prepared using the solvent casting technique according to Table 1. OLP/CMC film-forming solutions were obtained by dissolving CMC and OLP in distilled water with heating at 80 °C for 30 min. After that, glycerol (30% (*w*/*w*) total solid weight) and cinnamon essential oil (EO) (0.02% *v*/*v*, based on film-forming solution) were added to the mixture and homogenized at 15,000 rpm for 2 min (PT-1200C, KINEMATICA AG, Malters, Switzerland). EO concentration was based on the result of minimum inhibition concentration (MIC) obtained for test fungi; 10^6^ CFU/mL of *Aspergillus niger* KCCM32318 and *Trichoderma reesei* ATCC56765, using the agar dilution method [28] (Figure S1). Film-forming solutions were degassed using an ultrasonic bath (JAC-5020, KODO, Hwaseong, Korea), and 100 mL volume was poured into a mold (140 × 95 mm). After drying at 40 °C for 18 h, films were carefully peeled off and preconditioned in a desiccator at ambient and at 50% relative humidity until characterization.

### 2.4. Rheological Measurement of Film-Forming Solutions

The apparent viscosity and shear stress measurements of OLP/CMC composite solutions at different shear rates were measured using a Brookfield viscometer (DV-II + PRO, Middleboro, MA, USA) equipped with a cylindrical spindle (*#*40). Measurements were carried out at 25 °C, and data were fitted into the Power-law model (Equation (1)).

(1) $$\tau =K\gamma^{n}$$

where τ is the shear stress, *K* is the flow consistency index, γ is the shear rate, and *n* is the flow behavior index.

### 2.5. Characterization of OLP/CMC Composite Films

#### 2.5.1. FTIR Structural Characteristics 

Fourier transform infrared spectroscopy (FT-IR) analysis of films was performed using a PerkinElmer FTIR spectrophotometer (Frontier, Billerica, MA, USA) equipped with an attenuated total reflectance (ATR) module. Spectra readings between 400 and 4000 cm^−1^ at a resolution of 4 cm^−1^ were taken. 

#### 2.5.2. Thickness, Color Properties, and Transmittance

The thickness of each film was measured using a digimatic micrometer (NR 293-244-30, Mitutoyo, Kawasaki, Japan). The surface color values (L*, a*, and b*) of films were measured using a Chroma Meter (CR-300, Minolta Co., Osaka, Japan). The whiteness index (WI) of films was calculated using Equation (2). The light transmittance of films (30 × 10 mm) was measured by a UV-vis spectrophotometer (Shimadzu Co. UV-2550, Tokyo, Japan) at a 200–800 nm wavelength range. The opacity value was recorded at 600 nm and calculated using Equation (3); *x* is the film thickness (mm).

(2) $$\mathrm{WI}=\sqrt{{\left(100-L^{*}{)}^{2}+(a^{*}\right)}^{2}+{\left(b^{*}\right)}^{2}}$$

(3) $$\mathrm{Opacity}\;(A\;{\mathrm{mm}}^{-1})=\frac{{\mathrm{Abs}}_{600}}{x}$$

#### 2.5.3. Moisture Content Water Solubility and Contact Angle

Composite films (50 × 20 mm) were weighed (*W_o_*) and dried to a constant weight (*W*_1_) at 105 °C, and moisture content (MC) was calculated using Equation (4). Dried films were submerged in distilled water for 24 h at room temperature to determine water solubility (WS). The undissolved films were dried to constant weight (*W*_2_) at 105 °C. The WS was calculated using Equation (7) [29].

(4) $$\mathrm{MC}\;(\%)=\frac{W_{o}\times W_{1}\;}{W_{o}}\times 100$$

(5) $$\mathrm{WS}\;(\%)=\frac{W_{1}\times W_{2}\;}{W_{1}}\times 100$$

The films’ water contact angle (CA) was tested using a CA analyzer (DSA100, Kruss Scientific instrument, Hamburg, Germany) with a ±0.1° resolution. A 2 μL drop of ultra-pure water was placed on the film’s surface using a micro-syringe, and images were taken at 3, 6, and 9 s [10]. The CA value (*θ*) represents the mean average angle of the right and left angles of triplicate measurements per film sample.

#### 2.5.4. Oxygen and Water Barrier Properties

The oxygen permeability (OP) of films was determined as described by Zhang et al. [29]. Films were sealed to the top of an open vial (57 × 28 mm) containing three grams of deoxidizer consisting of iron powder, activated carbon, and sodium chloride (0.5:1.0:1.5) and placed in a desiccator containing saturated barium chloride solution (90%) at 25 °C. The weight of the vials was recorded after 48 h, and OP was calculated using Equation (6).

(6) $$\mathrm{OP}=\frac{∆M\;\times x\;}{A\times t}$$

where ∆M is the mass change of vial (g), *x* is the film thickness (m), A is the exposed area of the film (m^2^), and t (s) is the equilibrium time. 

The films’ water vapor permeability (WVP) was determined using a previous method [30]. A calcium chloride pellet was added into an open-top vial sealed with films. After weighing, vials were placed in a desiccator containing distilled water at 25 °C and weighed every 12 h for 6 days. The WVP was calculated using Equation (7).

(7) $$\mathrm{WVP}=\frac{∆W\times x\;}{∆t\times A\times ∆P}$$

where ∆W is the weight change of vial (g), *x* is the film thickness (m), ∆t is the time duration (s) for weight change, A is the exposed area of the film (m^2^), and ΔP (2339 Pa at 20 °C) is the vapor partial pressure.

#### 2.5.5. Mechanical Properties

The tensile strength (TS) and elongation at break (EB) of films (40 × 10 mm) were determined using a QMESYS Universal Material Testing Machine (QM100s, 1.96 kN, Komachine, Gyeonggi-do, Korea). The gauge distance and crosshead speed were set at 20 mm and 10 mm/s, respectively. The TS (MPa) and EB (%) results were computed by the QM-Pro Software.

#### 2.5.6. Thermal Stability Analysis

The thermal stability of the films (10 mg) was analyzed using an auto-thermogravimetric analyzer (Q500, TA Instruments, USA), and conditions as described in Section 2.5. Film samples were heated from 30 to 600 °C at a heating rate of 10 °C/min under a nitrogen atmosphere (20 mL/min). The TGA and DTG data were analyzed using Trios v4.5A software (TA Instruments, New Castle, DE, USA).

#### 2.5.7. Antioxidant Activity

ABTS radical scavenging (ABTS-RSA) and ferric reduction power (FRAP) assays evaluated the antioxidant activities of films. For this purpose, 0.1 g of each film was dissolved in 10 mL of distilled water by stirring overnight before being centrifuged to obtain a clear solution. For ABTS, 50 μL of the film solution (sample) or distilled water (blank) was reacted in the dark with 950 μL of ABTS radical cation solution for 30 min. ABTS cation solution consisted of 7 mM ABTS and 2.45 mM potassium persulfate in 10 mL of distilled water. The solution’s absorbance (734 nm) was adjusted to 1.0 with DW. The sample (A_1_) and blank (A_0_) absorbance was measured at 734 nm using a UV spectrophotometer, and ABTS-RSA was calculated using the following Equation (8).

(8) $$\mathrm{ABTS}-\mathrm{RSA}\;(\%)=\left[\left(\frac{A_{0}-A_{1}}{A_{0}}\right)\times 100\right]$$

For FRAP assay, 100 μL of the film solution (sample) or distilled water (blank) was reacted in the dark with 900 μL of FRAP reagent for 30 min. The FRAP reagent was prepared with 300 mM acetate buffer (pH 3.5), TPTZ (2,4,6-Tris(2-pyridyl)-s-triazine) solution (10 mM) in HCl (40 mM), and ferric chloride solution (20 mM) in HCl (40 mM) at a ratio of 10:1:1, respectively. The absorbance of the sample (A_1_) and blank (A_0_) was measured at 593 nm, and the results were expressed as corrected absorbance (abs).

### 2.6. Packaging Application of OLP/CMC as a Coating for Cherry Tomatoe Preservation

#### 2.6.1. Coating and Storage of Tomatoes

Cherry tomatoes were obtained from a local mart in Daegu, Korea. Tomatoes were sorted for visually similar sizes and redness, and damaged or mold-infected tomatoes were removed. Thereafter, tomatoes were washed with tap water, dried in a sterile air chamber, and precooled at 4 °C. Tomatoes were coated by immersing for 2 min in the film-forming solutions prepared in Section 2.3 and air-dried in a laminar flow cabinet. Tomatoes immersed in distilled water were denoted as the control sample. Tomatoes were stored in an incubator (LI-IL060, LKLAB, Namyangju, Korea) at 30 °C for 14 days.

#### 2.6.2. Quality Assessments during Storage

The percentage change in weight and firmness of stored cherry tomatoes was determined as described in our previous study [31]. Weight measurement was performed using a laboratory digital weighing balance (Mettler Toledo, CH/PL 3002), and pH and total soluble solid content (TSS) were measured using a digital pH meter (Mettler-Toledo AG8603, Schwerzenbach, Switzerland) and a pocket refractometer (PAL-1, Atago Co., Ltd., Tokyo, Japan). Firmness was measured by compression test to 50% penetration using a Rheometer (Compac-100D, Sun Scientific Co., Tokyo, Japan) equipped with a 20-mm cylindrical probe. Probe head speed and maximum force were set at 60 mm/min and 10 kg, respectively. Firmness (kg) was presented as the average value of six measurements for each treatment. The tomatoes’ color values (L*, lightness, a* red-green coordinate, and b* yellow-blue coordinates) were measured using a CR-300 Chroma meter (Minolta Co., Osaka, Japan). The color attributes of tomatoes were presented by redness value (Equation (9)) and color change (ΔE) (Equation (10)).

(9) $$\mathrm{Redness}=\frac{a^{*}}{b^{*}}$$

(10) $$\Delta E=\sqrt{{\left(L_{2}-L_{1}{)}^{2}+(a_{2}-a_{1}\right)}^{2}+{\left(b_{2}-b_{1}\right)}^{2}}$$

#### 2.6.3. Visual Quality

The visual appearance of tomatoes was obtained using an industrial camera (DFK 31AF03; Theimagingsource, Bremen, Germany), and the visual quality score was rated using a 9-point scale, where 9 denotes excellent and fresh appearance, 7 denotes good, 5 denotes fair (limit of marketability), 3 represents fair (useable but not saleable), and 1 indicates that the product is bad and unusable [32]. Fifteen panelists consisting of Graduate Students of Food Application Majors, who were familiar with cherry tomato quality, were used for this test. 

### 2.7. Statistical Analysis

Experimental measurements were taken in a minimum of triplicates (except when stated otherwise), and data analysis was performed using SPSS 20.0 software (IBM Inc., Chicago, IL, USA). Statistical differences (*p* < 0.05) between means were determined by analysis of variance and Duncan’s multiple ranges tests.

## 3. Results

### 3.1. Rheological Properties of Film-Forming Solutions

**Figure 1:**
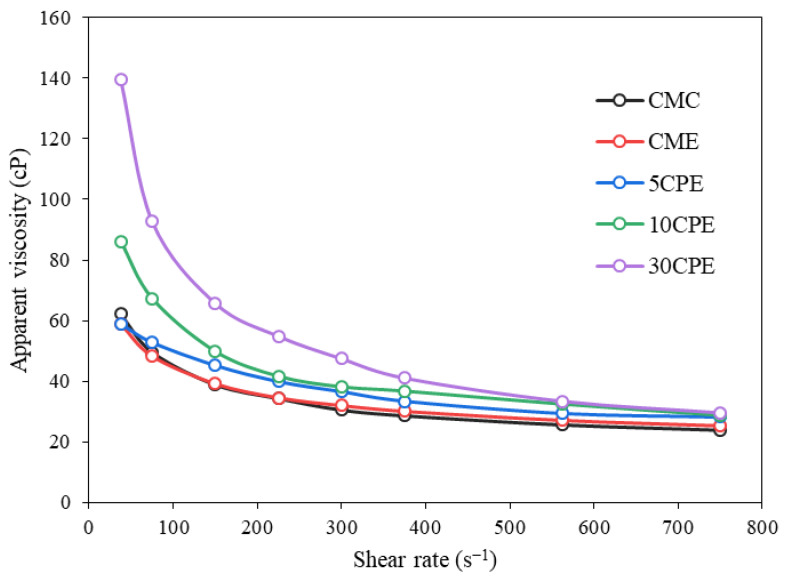
Apparent viscosity of OLP/CMC film-forming solutions at different shear rates.

The rheological characteristics of film-forming solutions influence film formation and the important properties of films [33]. The apparent viscosity plot at different shear rates is presented in Figure 1, and Power-law model indices were used to explain film-forming solutions’ flow behavior. All film-forming solutions exhibited a shear-thinning rheological behavior, with apparent viscosity decreasing with increasing shear rate. A similar rheological pattern has been observed for other common film-forming solutions, including carrageenan, guar, and xanthan gum [34]. Moreover, the apparent viscosity and flow for consistency coefficient (*K*) increased with the concentration of OLP, which is attributed to the formation of entangled networks induced by the increasing number of hydrogen bonds between OLP and CMC molecules [35]. All film-forming solutions exhibited non-Newtonian pseudoplastic fluid behavior (*n* < 1), having flow behavior index (*n*) values ranging from 0.478 to 0.743 (*r^2^* = 0.998 to 1.000) (Table 2). Notably, the differences in this value could relate to potential differences in their corresponding film properties [33].

### 3.2. FT-IR Structural Characteristics of Films

**Figure 2:**
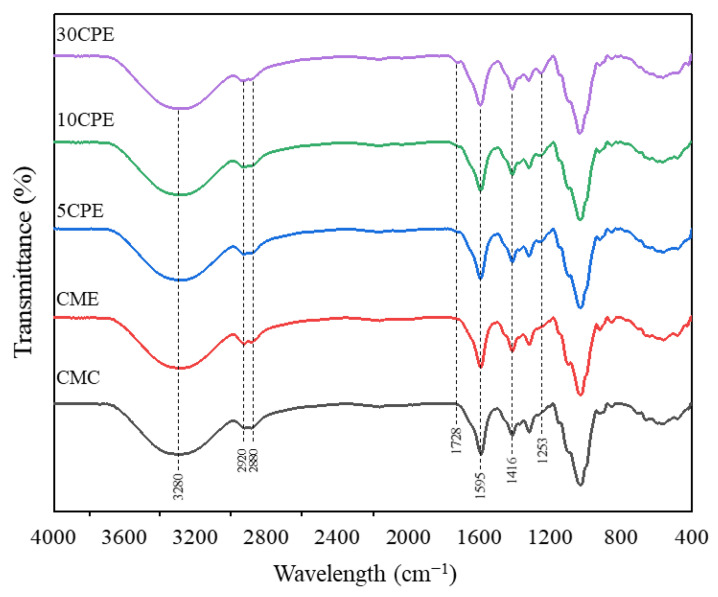
FT-IR spectra of composite OLP/CMC films.

The FT-IR spectra were used to observe major structural groups in the film samples (Figure 2). All films showed broad absorption peaks at around 3273–3289 cm^−1^, corresponding to the –OH stretching vibration of polysaccharides. In addition, the broad and shoulder peaks at 2920 and 2880 cm−1 were attributed to the C–H stretching vibrations of the polysaccharides [36]. The peaks at 1728 cm^−1^ and 1253 cm^−1^, which increased in intensity with an increase in OLP concentration in the film, are attributed to the presence of the esterified carboxyl group (C=O) in OLP, typical of pectic polysaccharides [37]. The absorption band around 1590–1596 cm^−1^ and 1416 cm^−1^ corresponds to the symmetrical and asymmetrical vibrational stretching of OLP and CMC carboxyl groups in their carboxylate anions (COO–) form [38]. Notably, positional shifts in the peak of –OH at 3289 to 3273 cm^−1^,and the slight shift in the peak of –COOH at 1590 to 1596 cm^−1^ in CPE biocomposite films after the addition of OLP confirmed intermolecular hydrogen bond interaction between OLP and CMC [39]. Other peaks within the spectral region of 1200 to 1000 cm^−1^ are related to asymmetric vibrations of glycosidic linkages (C–O–C and C–O), typical of polysaccharide-based films [29].

### 3.3. Color and Light Barrier Properties of Films

**Figure 3:**
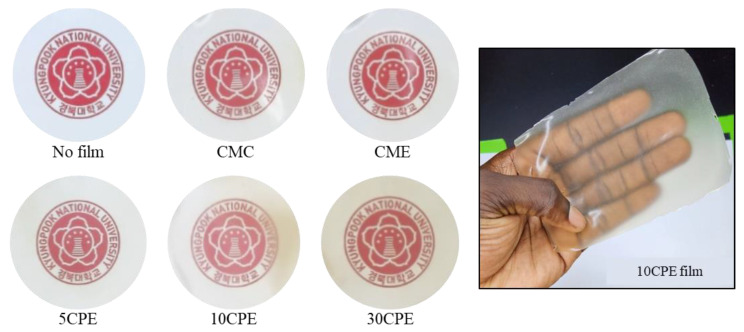
Visual appearance of OLP/CMC biocomposite films.

**Figure 4:**
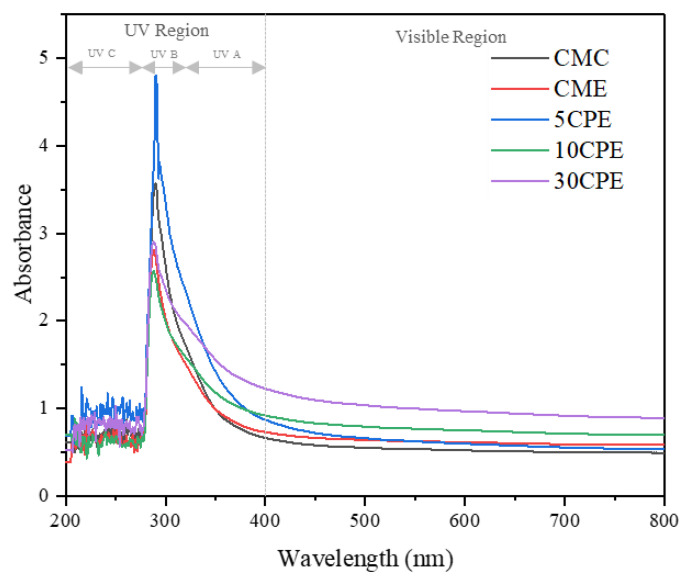
UV-vis light transmittance properties of films at 200–600 nm wavelength range.

The appearance of films and the UV-transmittance properties of films are shown in Figure 3 and Figure 4, respectively. The visual appearance of the films used for food packaging influences consumers’ perception of the product because it influences visibility. All films showed a smooth appearance and good flexibility, which increased with OLP composition. In addition, the brown surface color of films increased with an increase in OLP. The UV/light barrier property of films was evaluated in the wavelength range of 200 to 800 nm (Figure 4) All films showed good UV barrier properties, especially in completely blocking UV-B and UV-C radiations. However, the restriction of light transmission (opacity) was more evident in OLP/CMC composite films (5CPE < 10CPE < 30CPE) than in CMC-based films (Table 3), which is attributed to the characteristic color of OLP powder [13]. In addition, the lightness (L* value) and the whiteness index (WI) of composite films decreased gradually with an increase in OLP, whereas a* and b* values increased significantly (*p* < 0.05) (Table 3). The variation in color values results from the natural color of OLP, which could be due to the presence of pigment or phenolic compounds co-extracted with the mucilage [40]. The opacity and WI values of OLP/CMC films reported in this study are in similar ranges to polysaccharide-based films [36,38]. Transparent and colorless packages are necessary to avoid consumers’ visual misjudgment of foods, but slightly opaque packages are preferred to prevent deterioration and spoilage associated with photo-oxidation in light-sensitive food materials [29,36]. Notably, the inclusion of essential oil (EO) showed an insignificant influence on the light transmittance and color properties of films (*p* < 0.05), which could be due to its low content in the film-forming solutions (Figure 4 and Table 3).

### 3.4. Thickness and Mechanical Properties of Films

**Figure 5:**
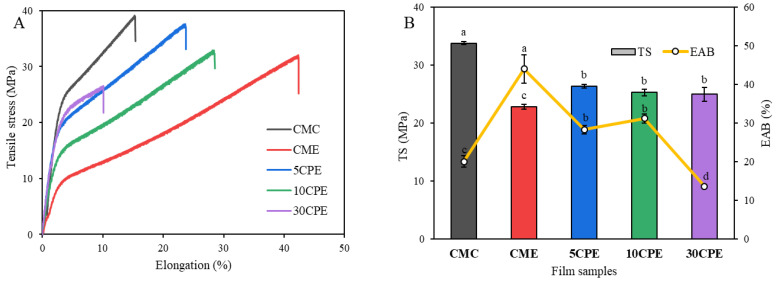
Mechanical properties of films. Tensile stress versus strain curve (**A**), and tensile strength and elongation at break (**B**) of films. Values represent mean ± SD (*n* = 3), and different superscripts denote significant differences (DMRT, *p* < 0.05).

The thickness and mechanical properties of films are presented in Table 3 and Figure 5, respectively. The thickness of composite films ranged from 97.67 to 150.33 μm and decreased significantly with increasing content of OLP in the film-forming solutions (Table 3). However, EO inclusion in CMC did not influence the thickness of the CME film (*p* < 0.05). Because the same solid contents and volume of film-forming solutions were used to prepare the films, differences in thickness could be attributed to inconsistent levels of shrinkage in films [29]. The higher thickness of CMC films could be attributed to swelling by absorption of moisture [41]; however, increasing OLP in composite films resulted in reduced retention of moisture (Table 4). 

The mechanical properties of tensile strength (TS) and elongation at break (EAB) of films are presented in Figure 5. The TS of CMC film (33.78 MPa) was significantly higher (*p* < 0.05) than those of biocomposite films (CPE, 24.97–26.34 MPa) and CMC film containing EO (CME, 22.81 MPa). Notably, the inclusion of EO reduced the tensile strength of all films, as observed in CME; however, the subsequent addition of OLP slightly increased the TS of biocomposite films (CPEs) due to increased hydrogen bonding interactions between OLP/CMC polymer matrixes and enhanced interfacial adhesion in film structure [29]. In contrast, the flexibility of CMC film (20.00%) significantly increased with the incorporation of EO, with CME having an EAB value of 43.95%. The mechanism of EO in improving EAB could be attributed to the enhanced intermolecular spacing and chain mobility between polymeric chains (reduced chain interactions), which resulted in an expanded structure and volume of the film [5,19]. Similar trends have been reported for polysaccharide-based films containing essential oils [20,34]. Moreover, the subsequent addition of OLP up to 10% w/w of CMC slightly reduced EAB. At a higher concentration of 30% OLP, the EAB of 30CPE film decreased considerably to 13.60% due to the formation of non-homogenous film structures. The lower tensile strength of OLP/CMC films relates to the weak strength of OLP polymer, causing a less compact molecule film structure [9]. Moreover, the TS and EAB of films are influenced by the viscosity of the film-forming solution [42], which agrees with the result of this study.

### 3.5. Water Sensitivity

The moisture content, water solubility, and contact angle of films are presented in Table 4. Moisture content (MC) varied (7.61–9.39%) significantly among film samples (*p* < 0.05). The variations and lower MC in 10CPE and 30CPE films could be attributed to competitive hydrogen bonding between OLP and CMC polymer chains, which reduced the interaction between water molecules and the hydroxyl group of polymers, leading to lowered water absorption into the film voids [37]. All films showed good water solubility (83.30–95.96%), in agreement with previous reports [42]. However, the decrease in the water solubility of films containing EO may be due to the disruption of the intermolecular chain interaction of polymer matrix by the hydrophobic oil, which led to the cohesiveness of the polymer matrix [9]. In addition, the gradual increase in WS of biocomposite films upon the subsequent addition of OLP might have resulted from the increase in the hydroxyl group contributed by OLP, which enhanced film interaction with water molecules [42]. 

The water contact angle (CA) indicates the film’s surface affinity to water [38]. The CA of CMC films increased with EO due to the oil’s hydrophobic nature (Table 4). A similar increase in the CA of polysaccharide-based films by incorporating essential oils has been observed [43]. However, the reduction in CA of biocomposite films with increasing OLP content could be attributed to the hydrophilicity nature of the mucilage polysaccharides [5,44].

### 3.6. OP and WVP of Films

**Figure 6:**
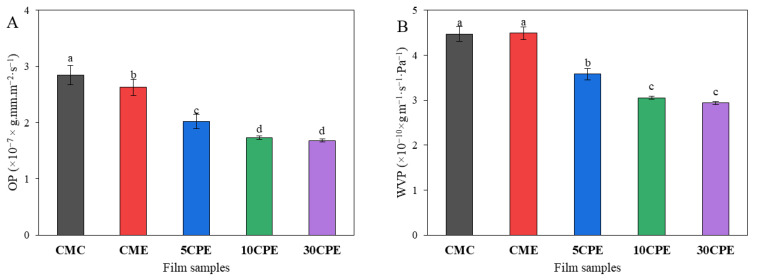
Oxygen (**A**) and water vapor permeability (**B**) of films. Values represent mean ± SD (*n* = 3), and different superscripts denote significant differences (DMRT, *p* < 0.05).

The oxygen (OP) and water vapor permeability (WVP) properties of films intended for food packaging influence their preservative efficacy. The OP and WVP of films varied significantly from 1.68 to 2.85 × 10^−7^ × g·mm·m^−2^·s^−1^ (Figure 6A) and 2.94 to 4.48 × 10^−10^ × g m^−1^·s^−1^·Pa^−1^ (Figure 6B), respectively. The polymer components of film predict their barrier properties. Notably, the moderate addition of OLP (up 10%) increased the oxygen barrier and water vapor properties of OLP/CMC biocomposite films (CPE) compared to films fabricated with CMC alone. This could be attributed to the compact network of hydrogen bonding formed between OLP and CMC, which restricted the easy diffusion of oxygen and water vapor [45]. In addition, the higher water vapor barrier properties observed in biocomposite films (CPE), compared to CMC films, could have resulted from the tight-filling of the micro paths in the film network by OLP molecules [39]. However, further incorporation of OLP resulted in insignificant enhancements in barrier properties (Figure 6). Kang et al. [35] reported similar findings upon incorporating okra pod mucilage up to 4% in PVA films. Low water vapor and oxygen permeability are preferred for fresh produce packaging due to their high rate of respiration and transpiration and other chemical reactions associated with fluid and water-soluble nutrient losses [5]. The OP [45] and WVP [33] reported in this study are better than or in a similar range to other studies. OLP/CMC biocomposite films generally exhibited good OP and WVP applicable for food packaging purposes.

### 3.7. Thermal Properties of Films

**Figure 7:**
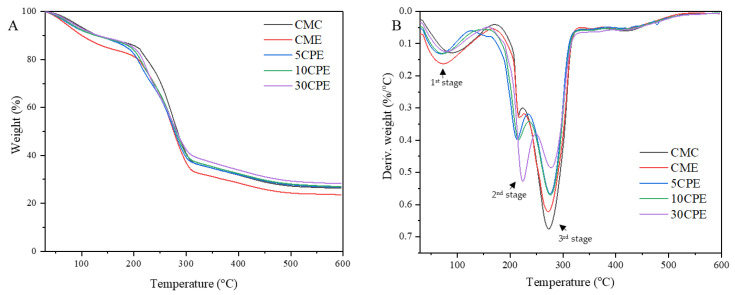
Thermal properties; TG (**A**) and DTG (**B**) curve of films.

The thermal stability of films was evaluated using their thermogravimetry (TG) and derivative thermogravimetry (DTG) curves (Figure 7). Three thermal degradation stages were recorded (Table 5). The first stage, observed around 100 °C, showed a similar weight loss in the range of 10–16% due to the evaporation of free and bound moisture contained in the film samples [9]. The second stage occurred between 180–250 °C, and variations in weight loss (7–23%) were observed according to the content of OLP. Weight losses at this stage are associated with the degradation of mucilage, pectin, and glycerol plasticizer in the film [41,46]. Thus, higher weight loss occurred in CPE films as the content of OLP increased in the film matrix. The final stage accounted for the highest weight loss ranging between 35 and 53%, due to the degradation of CMC [42]. The weight loss at this stage was inversely proportional to the OLP content, in that films containing a higher concentration of OLP showed lesser weight loss. Furthermore, the broadest peak in the DTG curve, representing the maximum degradation temperature (T_max_), occurred at 269–282 °C. CPE films showed a higher maximum degradation temperature (>280 °C) than CMC and CME films, indicating that OLP improved the thermal stability property of OLP/CMC biocomposite films.

### 3.8. Antioxidant Activity of Films

**Figure 8:**
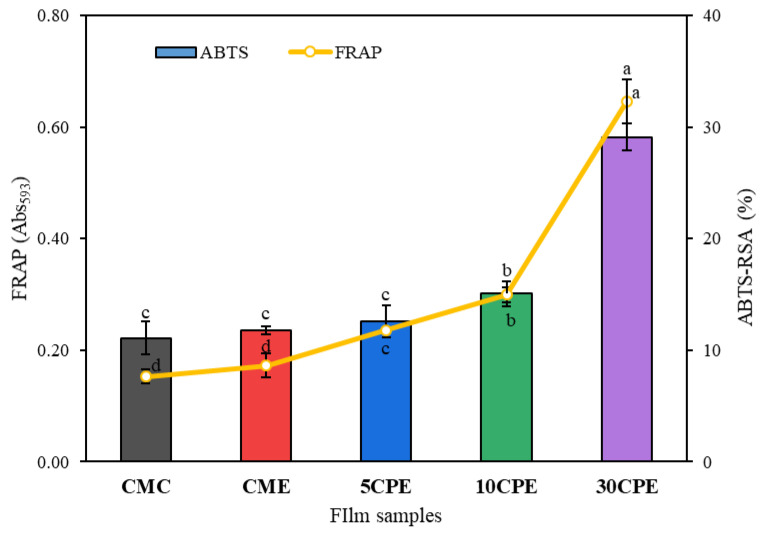
FRAP and ABTS antioxidant activities of films. Values represent mean ± SD (*n* = 3), and different superscripts denote significant differences (DMRT, *p* < 0.05).

OLP/CMC biocomposite (CPE) films showed a significant (*p* < 0.05) increase in ABTS (11.11–29.10%) and FRAP (0.15–0.65) antioxidant activity as the content of OLP increased (Figure 8). The antioxidant activity of okra leaf polysaccharides (OLP) has been previously reported [13]. Moreover, the inclusion of essential oil showed no improvements in the antioxidant activities of films, which may be due to its low content in the film’s composition. The antioxidant properties of OLP may be attributed to their hydrogen ions (H^+^) donating ability, and the presence of polyphenolic compounds [40]. The antioxidant potency of OLP/CMC films provides additional nutritional benefits besides packaging functions, when films are consumed along with food products. 

### 3.9. Application of OLP/CMC for Cherry Tomatoe Preservation

OLP/CMC film-forming solutions were applied as a coating on cherry tomatoes to evaluate their food packaging efficiency. Changes in quality parameters, including weight loss, firmness, color properties, pH, and visual quality scores, were measured during 14 days of storage at 30 °C. Storage temperature at 30 °C stimulated local market conditions [47] and observed real-time deterioration in cherry tomatoes.

#### 3.9.1. Changes in Weight, Firmness, and Color

**Figure 9:**
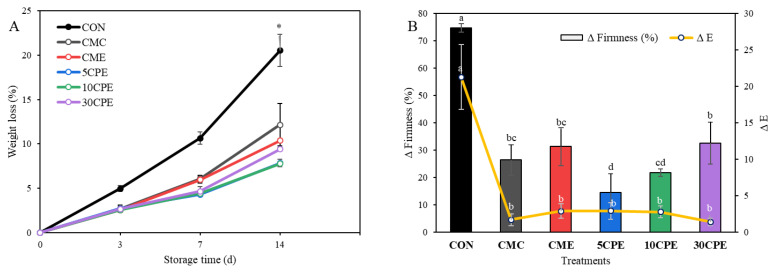
Percentage weight loss (**A**) and changes in color (∆E) and percentage change in firmness (**B**) of uncoated and OLP/CMC-coated cherry tomatoes during storage at 30 °C for 14 days. Values represent mean ± SD (*n* = 3), and different superscripts or ‘*’ denotes significant differences (DMRT, *p* < 0.05).

The changes in the weight of uncoated and coated tomatoes, presented as percentage weight loss, are shown in Figure 9A. Gradual weight loss in all samples due to transpiration has been widely reported for stored cherry tomatoes [20,37]. At the end of 14 days of storage, weight loss in the untreated sample (CON) exceeded 20%. However, OLP/CMC coating significantly minimized weight loss (7.75–12.16%) during storage. Among all treatments, 5CPE and 10CPE coating presented significantly lower weight loss during storage (*p* < 0.05). Similar weight loss reduction in tomatoes coated with polysaccharide-based solutions has been reported [18].

Slight variations in the initial pH of tomatoes, attributed to product variety, have been previously reported in another study [21]. However, an increase in pH was recorded in all samples after storage (Table 6), typical of fruit and vegetable during storage [21]. 

Color change and firmness are common quality changes in stored tomatoes. More so, they are key qualitative parameters for consumer acceptability [18]. After the storage period, noticeable differences in color and firmness properties were observed, especially in uncoated tomatoes (Table 6). For instance, an increase in lightness (L* value) from 36.86 to 46.61, a reduction in redness index (a*/b*) from 1.02 to 0.65, and a large decrease in firmness (from 683.33 to 173.40 kg/cm^2^) were observed in uncoated tomatoes after the storage period. On the other hand, coated tomatoes showed notable retention of color parameters. The change in color (∆E) and firmness (∆firmness) are presented in Figure 9B. ∆E ranged from 1.39 to 21.26, while ∆firmness ranged from 14.49 to 74.62%. Color change in tomatoes is associated with an increase in carotenoids and anthocyanin pigments due to ripening or discoloration observed on the skin due to spoilage [22]. 

On the other hand, the reduction in firmness of tomatoes during storage is related to the degradation and changes in the cell wall compositions and biochemical components and is not limited to moisture loss [47]. In particular, the degradative activities of cell-wall enzymes such as polygalacturonase and pectin methylesterase are responsible for changes in the firm structure of fruits and vegetables. The retained firmness in OLP/CMC could be attributed to the active barrier properties of the thin-film layer formed by the coating solution, which retarded the rate of respiration and transpiration, preventing softening and controlling enzyme activities. In addition, the incorporation of essential oil in film-forming has been reported to contribute to firmness preservation by inhibiting the degradative activities of cell wall enzymes [21,47,48]. 

#### 3.9.2. Visual Appearance and Quality Score

**Figure 10:**
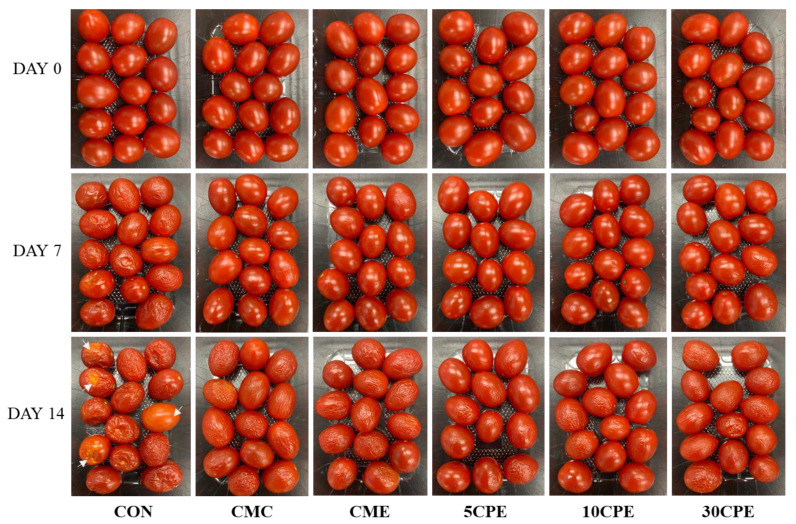
Visual appearance of uncoated and OLP/CMC-coated cherry tomatoes during storage at 30 °C for 14 days.

**Figure 11:**
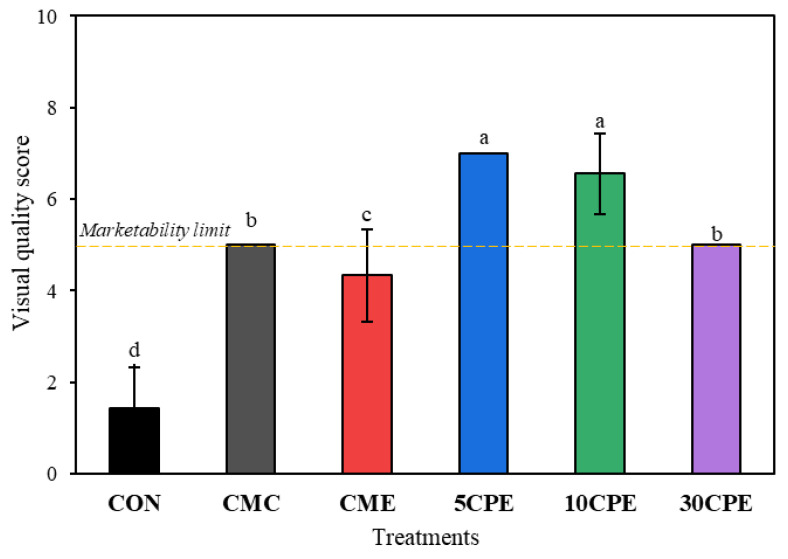
The visual appearance of uncoated and OLP/CMC-coated cherry tomatoes during storage at 30 °C for 14 days. The yellow dashed-line denotes the limit of marketability. Values represent mean ± SD (*n* = 15), and different superscripts indicate significant differences (DMRT, *p* < 0.05).

Figure 10 shows the visual appearance of uncoated and coated tomatoes during storage, and the visual quality scores after the storage period are presented in Figure 11. After 7 days of storage, visible shrinkage was observed in untreated tomatoes, while coated samples maintained a fresh appearance. However, after 14 days, spoilage characterized by soft spots, juice linkages, and yellow discoloration was observed in uncoated tomatoes (white arrows, Figure 10). Slight shrinkage was also observed in CMC-, CME-, and 30CPE- coated tomatoes, attributed to moisture loss, but no visible discoloration. Improved quality preservation in 5CPE- and 10CPE-coated tomatoes could be related to their better film properties. 

The visual score represents a cumulative evaluation of the quality in terms of the expected appearance, color, texture, or form perceived by the consumer, which often influences the economic value and the decision of the consumer to purchase the product. Among all samples, only 5CPE- (7.00) and 10CPE- (6.56) coated tomatoes showed high visual quality scores exceeding the limit of marketability (Figure 11). CMC- and 30CPE-coated samples with visual quality scores of 5.00 showed fair ratings and were within market limits. However, uncoated tomatoes were judged as unusable, indicated by their low scores (1.44). Similar reports on the maintenance of visual quality in fruits with polysaccharide-based coatings have been reported in previous studies [32,49,50]. Overall, our results reflected that OLP/CMC was effective in preserving the quality and extending cherry tomatoes’ shelf life, compared with other previous studies (Table S1).

## 4. Conclusions

This study successfully used mucilage polysaccharides and CMC derived from okra plant wastes to prepare edible food packaging. OLP/CMC biocomposites were characterized for their film-forming properties and applied as an edible food coating to preserve cherry tomatoes. The incorporation of cinnamon essential oil slightly influenced specific properties of the film, such as mechanical flexibility. Relatively notable improvements in the properties of OLP/CMC biocomposite films were observed upon increasing the content of OLP, up to 10% *w*/*w* based on CMC, owing to enhanced intermolecular bonding between the composite polymers. In particular, OP and WVP barrier properties, essential for fresh produce packaging, were substantially enhanced, which was reflected correspondingly in their preservative efficiency on cherry tomatoes during a 14 day storage period. After the storage period, coated tomatoes showed comparably better quality and were within marketable limits, whereas severe quality deteriorations occurred in uncoated tomatoes. This study offers a practical route for converting accumulated waste from okra plants into value-added biopolymers, which is applicable for food packaging applications.

## Figures and Tables

**Table 1:** OLP/CMC biocomposite films composition.

Films	Composition of Film-Forming Solution (g/100 mL)		
	CMC	OLP	EO
CMC	2	0	0
CME	2	0	0.02
5CPE	1.9	0.1	0.02
10CPE	1.8	0.2	0.02
30CPE	1.4	0.6	0.02

**Table 2:** Rheological model indices for OLP/CMC film-forming solutions.

Model Indices	*K*	*n*	*r* ^2^
CMC	1.731	0.699	0.999
CME	1.380	0.743	1.000
5CPE	1.872	0.711	0.999
10CPE	2.641	0.666	0.998
30CPE	9.154	0.478	0.999

*K* and *n* are the Power law model constants described in Equation (1), while *r*^2^ is the model correlation coefficient.

**Table 3:** Thickness and color properties of films.

Films	Thickness (μm)	L*	a*	b*	WI	Opacity (A mm^−1^)
CMC	150.33 ± 17.93 ^a^	89.93 ± 1.72 ^a^	−0.73 ± 0.19 ^d^	9.75 ± 0.64 ^b^	85.95 ± 1.67 ^ab^	3.51 ± 0.15 ^d^
CME	151.67 ± 8.39 ^a^	90.85 ± 0.67 ^a^	−0.92 ± 0.06 ^d^	10.10 ± 1.04 ^b^	86.34 ± 1.22 ^a^	4.09 ± 0.13 ^d^
5CPE	119.33 ± 3.51 ^b^	86.87 ± 1.53 ^b^	−0.48 ± 0.10 ^c^	11.27 ± 1.60 ^b^	82.68 ± 2.11 ^ab^	5.12 ± 0.37 ^c^
10CPE	104.33 ± 3.51 ^bc^	86.64 ± 1.78 ^b^	−0.23 ± 0.02 ^b^	11.52 ± 1.98 ^b^	82.35 ± 2.64 ^b^	7.27 ± 0.30 ^b^
30CPE	97.67 ± 1.15 ^c^	80.94 ± 1.78 ^c^	0.16 ± 0.19 ^a^	19.00 ± 1.30 ^a^	73.09 ± 2.17 ^c^	10.07 ± 0.51 ^a^

Values represent mean ± SD (*n* = 3), and different superscripts in the same column denote significant differences (DMRT, *p* < 0.05).

**Table 4:** Moisture content, water solubility, and contact angle of films.

	MC (%)	WS (%)	CA (°)
CMC	9.32 ± 0.10 ^a^	95.74 ± 1.38 ^a^	79.50 ± 1.70 ^c^
CME	9.39 ± 0.49 ^a^	83.30 ± 1.26 ^d^	97.85 ± 0.64 ^a^
5CPE	9.28 ± 0.14 ^a^	87.54 ± 0.31 ^c^	98.40 ± 1.27 ^a^
10CPE	8.07 ± 0.23 ^b^	90.87 ± 1.40 ^b^	89.95 ± 0.21 ^b^
30CPE	8.01 ± 0.59 ^b^	95.96 ± 1.04 ^a^	88.75 ± 1.14 ^b^

Values represent mean ± SD (*n* = 3), and different superscripts in the same column denote significant differences (DMRT, *p* < 0.05).

**Table 5:** Weight loss at stages of thermal degradation and maximum degradation temperature of films.

Films	Mass Loss (%)			Residue (%)	DTG (T_max,_ °C)
	1st Stage	2nd Stage	3rd Stage		
CMC	12.19	7.49	52.86	27.46	275.94
CME	15.48	9.15	49.22	26.16	276.02
5CPE	10.30	18.42	41.95	29.34	280.61
10CPE	11.06	17.24	42.67	29.03	280.65
30CPE	11.30	23.01	35.84	29.85	281.34

**Table 6:** Color properties, pH, and firmness of uncoated and OLP/CMC-coated cherry tomatoes.

Storage	Treatments	L*	a*	b*	Redness (a*/b*)	pH	Firmness (kg/cm^2^)
0 d	CON	36.86 ± 0.56 ^a^	17.32 ± 0.59 ^a^	17.02 ± 0.50 ^a^	1.02 ± 0.06 ^a^	4.39 ± 0.01 ^c^	683.33 ± 42.52 ^a^
	CMC	35.94 ± 0.25 ^a^	16.36 ± 1.09 ^a^	15.77 ± 1.18 ^a^	1.04 ± 0.01 ^a^	4.43 ± 0.01 ^a^	621.67 ± 162.58 ^a^
	CME	36.13 ± 1.34 ^a^	15.01 ± 1.96 ^a^	15.93 ± 0.41 ^a^	0.94 ± 0.10 ^a^	4.31 ± 0.00 ^d^	582.75 ± 52.68 ^a^
	5CPE	36.24 ± 0.38 ^a^	17.42 ± 2.02 ^a^	16.35 ± 0.84 ^a^	1.06 ± 0.07 ^a^	4.38 ± 0.00 ^c^	630.00 ± 55.00 ^a^
	10CPE	36.89 ± 0.44 ^a^	17.42 ± 1.79 ^a^	16.76 ± 0.98 ^a^	1.04 ± 0.09 ^a^	4.40 ± 0.00 ^b^	605.00 ± 35.00 ^a^
	30CPE	36.03 ± 0.49 ^a^	17.03 ± 1.00 ^a^	16.69 ± 0.23 ^a^	1.02 ± 0.06 ^a^	4.40 ± 0.00 ^b^	668.33 ± 35.12 ^a^
14 d	CON	46.61 ± 2.17 ^a^	21.59 ± 3.43 ^a^	34.86 ± 8.34 ^a^	0.65 ± 0.25 ^b^	4.52 ± 0.01 ^d^	173.40 ± 3.57 ^d^
	CMC	36.12 ± 1.15 ^b^	16.82 ± 0.85 ^b^	16.59 ± 1.15 ^a^	1.02 ± 0.09 ^a^	4.58 ± 0.01 ^b^	457.90 ± 34.99 ^bc^
	CME	35.86 ± 0.75 ^b^	17.25 ± 1.44 ^b^	15.00 ± 1.31 ^a^	1.15 ± 0.09 ^a^	4.60 ± 0.01 ^a^	400.82 ± 40.46 ^c^
	5CPE	36.32 ± 0.81 ^b^	16.23 ± 2.00 ^b^	14.37 ± 1.82 ^a^	1.13 ± 0.06 ^a^	4.57 ± 0.01 ^ab^	538.73 ± 42.29 ^a^
	10CPE	36.52 ± 0.24 ^b^	16.85 ± 2.18 ^b^	14.89 ± 1.57 ^a^	1.13 ± 0.07 ^a^	4.57 ± 0.01 ^ab^	474.00 ± 8.32 ^b^
	30CPE	36.42 ± 0.89 ^b^	16.44 ± 0.54 ^b^	15.91 ± 0.67 ^a^	1.03 ± 0.08 ^a^	4.56 ± 0.01 ^c^	450.93 ± 51.04 ^bc^

Values represent mean ± SD (*n* = 3), and different superscripts in the same column denote significant differences (DMRT, *p* < 0.05).

## Data Availability

The data presented in this study are available on request from the corresponding author.

## Supplementary Materials

The following supporting information can be downloaded at: https://www.mdpi.com/article/10.3390/polym14224884/s1, Figure S1: Minimum inhibition concentration (MIC) using the agar dilution method for test fungi (106). Aspergillus niger KCCM32318 (A) and Trichoderma reesei ATCC56765 (B); Figure S2: Process chart for isolation of cellulose from okra stalk powder and synthesis of carboxymethyl cellulose (CMC); Figure S3: FT-IR spectra (A) and XRD pattern of okra stalk powder (OKS), isolated cellulose (OKS-C), and synthesized OKS-CMC (B); Table S1. Comparison of preservative effects of CMC and their composite coatings on tomatoes. References [18,22,51,52,53,54,55] are cited in the supplementary materials.
