# Supplementary material for: Development and Characterization of Biocomposite Films Based on Polysaccharides Derived from Okra Plant Waste for Food Packaging Application
Source: Polymers (Basel). 2022 Nov 12;14(22):4884. doi: 10.3390/polym14224884 (PMC9692357; doi:10.3390/polym14224884)
Supplement: Supplementary file 1 [file polymers-14-04884-s001.zip › polymers-2022644-supplementary.pdf]

Supplementary file: **Development and Characterization of Biocomposite Films Based on Polysaccharides Derived from Okra Plant Waste for Food Packaging Application**

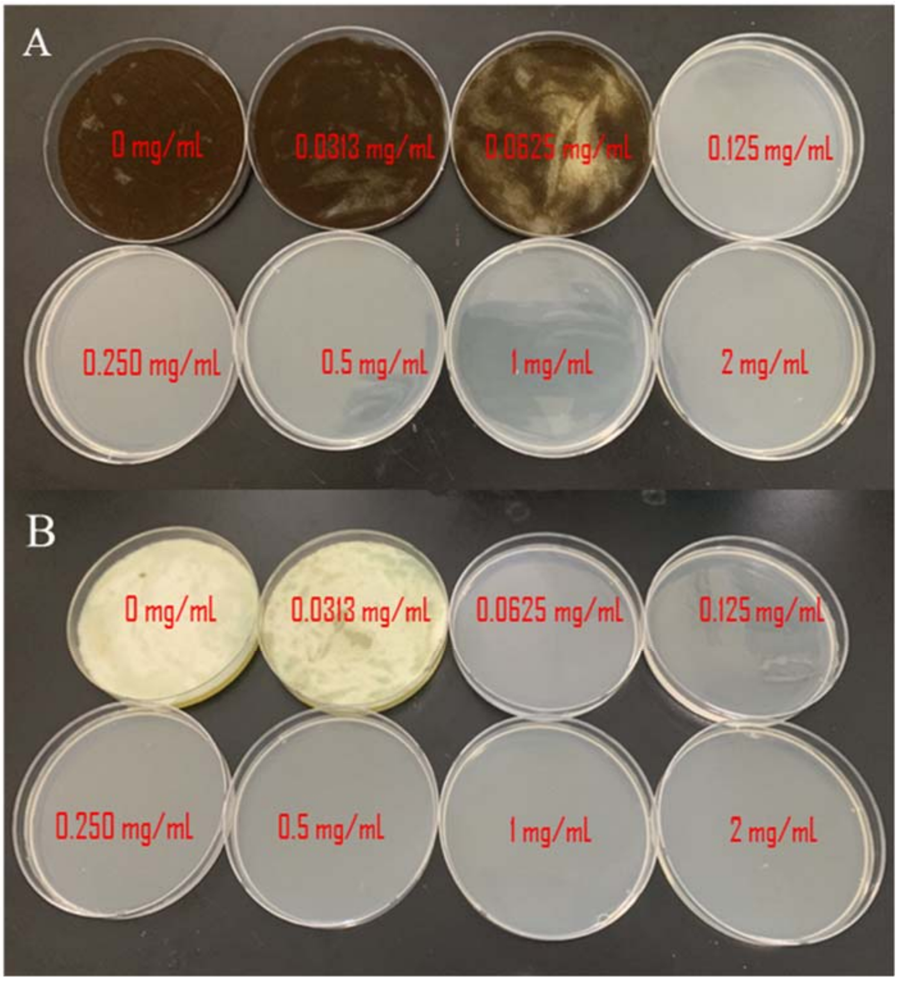

Figure S1. Minimum inhibition concentration (MIC) using the agar dilution method for test fungi (10^6). *Aspergillus niger* KCCM32318 (A) and *Trichoderma reesei* ATCC56765 (B).

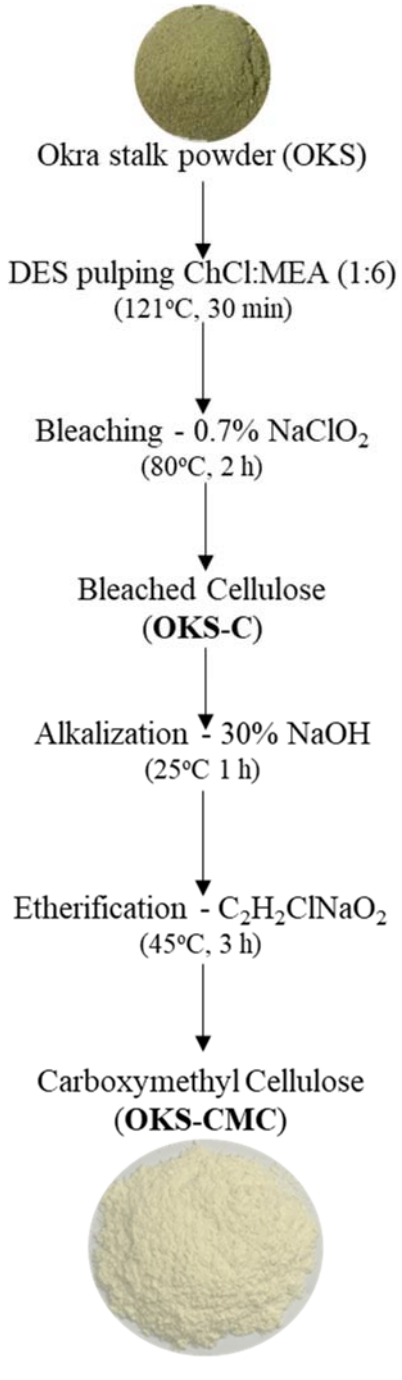

Figure S2. Process chart for isolation of cellulose from okra stalk powder and synthesis of carboxymethyl cellulose (CMC)

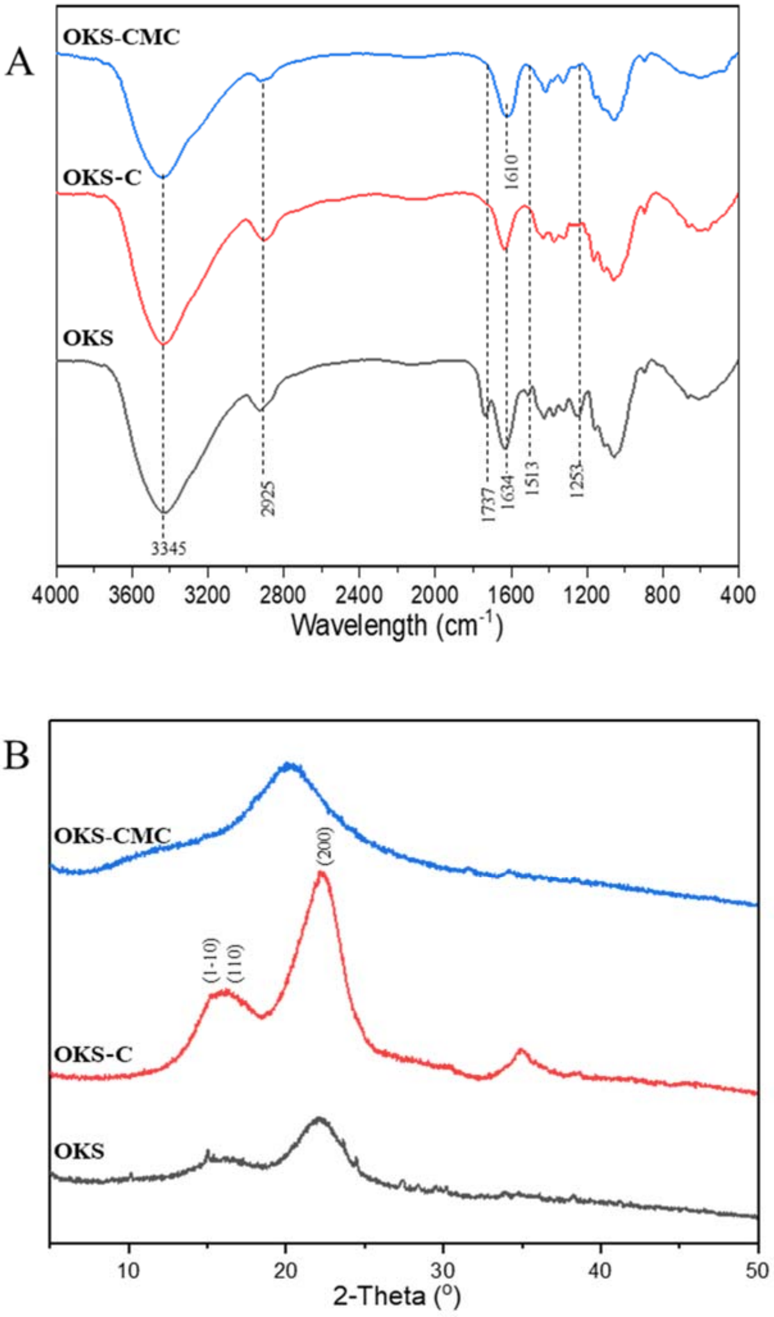

Figure S3. FT-IR spectra (A) and XRD pattern of okra stalk powder (OKS), isolated cellulose (OKS-C), and synthesized OKS-CMC (B).

Figure Discussion

The absorption peaks at 3445 cm^{-1} and 2925 cm^{-1} in the FT-IR spectra (Figure S3A) were attributed to O-H and C-H stretching vibrations, respectively. In OKS and OKS-C, the stretching vibration of carbonyl groups (C=O) of the acetyl group of hemicelluloses or the ester linkage in lignin were observed at peaks at 1737 cm^{-1}, 1513 cm^{-1}, and 1253 cm^{-1}. The absence of this peak in OKS-C proved that the lignin and hemicellulose had been removed [51,52]. The strong peak intensity at 1610 cm^{-1} in OKS-CMC, is due to the stretching vibrations of carboxylate anion's (COO-), indicating the

successful synthesis of the carboxymethyl substituents of CMC. Additionally, stronger peak intensity at 1610 cm^{-1} and diminished O-H peak at 2925 cm^{-1} in OKS-CMC compared to OKS-C further confirms the synthesis of CMC [51].

Following the isolation of cellulose from OKS and carboxymethylation of OKS-C, changes in crystallinity were visible in the XRD pattern (Figure S3B). The crystalline planes at 2θ 15.4°, 16.2° 22.1°, and 34.7° (displayed in OKS and OKS-C spectra represents 1-10), (110), (200), and (004), respectively, which are typical cellulose I patterns [51,53]. Additionally, OKS-C displayed greater crystallinity as a result of impurities being removed. Notably, the chemical modification of the cellulose to CMC resulted in the degradation of the crystalline peak at 22.1° in OKS-CMC [51].

Table S1. Comparison of preservative effects of CMC and their composite coatings on tomatoes

Film material	Food materials	Preservative Effects	Storage Condition	Reference
CMC	Cherry tomatoes	Lowered weight loss but no significant effect on firmness retention	20 d, RT	[54]
	Tomatoes	Reduced weight loss, color changes, and preserved firmness	15 d, 25°C	[55]
	Tomatoes	A slight reduction in weight loss and retained firmness	15 d, 25°C	[55]
	Tomatoes	Reduced weight loss and firmness loss	15 d, 25°C	[18]
**Composites**				
CMC + Gelatin	Tomato	Reduced weight loss and microbial growth	14 d, 25°C	[22]
CMC + *Osmunda japonica* polysaccharides	Tomato	Reduced weight loss, firmness loss, and inhibited decay	25 d, 25°C	[22]
CMC + Sodium alginate + chitosan biguanidine hydrochloride	Tomato	Reduced weight loss and extended shelf-life	20 d, RT	[22]
CMC + Okra leaf polysaccharides (OLP)	Cherry tomato	Retained visual appearance, firmness, and reduced weight loss. Inhibited decay	14 d, 30°C	This Study

RT: room temperature
